# Special Issue “Tumors of the Nervous System: New Insights into Signaling, Genetics and Therapeutic Targeting”

**DOI:** 10.3390/ijms23158700

**Published:** 2022-08-04

**Authors:** Sabrina Di Bartolomeo, Marco Segatto

**Affiliations:** Department of Biosciences and Territory, University of Molise, 86090 Pesche, IS, Italy

This Special Issue focused on the current understanding of signaling pathways as well as genetic and epigenetic features involved in the pathogenesis of brain tumors and other nervous system tumors, with emphasis on the development of novel therapeutic approaches aimed at improving the current standard of care.

Brain tumors and other nervous-system tumors belong to an extremely heterogeneous group of benign and malignant tumors characterized by different aggressiveness and variable clinical outcomes [1]. Among them, the most common primary brain tumors in adults are gliomas, meningiomas, and pituitary adenomas; medulloblastomas and gliomas are the most common brain tumors in children [1].

In this context, Haase et al. discussed the molecular features, development, and therapeutic perspectives on pediatric high-grade gliomas (pHGGs) arising in cerebral hemispheres [2]. Indeed, the revolutionary recent discoveries derived from genomic and epigenomic high-throughput profiling techniques led to the identification of prevalent molecular alterations in pHGGs and revealed a strong connection between epigenetic dysregulation and pHGG development. In particular, Haase et al. discussed the role of driving mutations and especially of epigenetic-disrupting mutations in pHGG onset, as well as the possibility of targeting these unique molecular vulnerabilities to design innovative tailored therapies for hemispherical pHGGs [2].

The current knowledge regarding the epigenetic mechanisms involved in medulloblastoma (MB) pathogenesis is described by Strejczek et al. MB is one of the most frequent and malignant brain tumors in children. Current therapies, despite being quite effective, unfortunately cause adverse effects that influence the central nervous system’s function, thus lowering the quality of life of patients. Epigenetic-based potential therapies for MB that could ensure a higher survival rate while maintaining a good quality of life are described in this review [3].

Epigenome deregulation plays a crucial role in several tumors besides MB. Notably, bromodomain and extraterminal domain (BET) proteins are epigenome readers of acetylated signals in histones and coactivators for oncogenic transcriptional networks [4]. BET proteins are thus promising targets for pharmaceutical intervention in cancer. Servidei et al. explored BET inhibition as an anticancer strategy in ependymoma stem-cell models. The BET inhibitor OTX015 reduced cell proliferation by inducing G0/G1-phase accumulation ad apoptosis in in vitro ependymoma models at clinically tolerable doses [5]. In detail, a p53-independent increase in p21 and p27 was observed upon OTX015 treatment, whereas proliferative signals mediated by STAT3 were decreased. Moreover, the up-regulation of apoptosis-related proteins correlated with cell-line drug sensitivity. OTX015 also improved survival in orthotopic ependymoma models [5]. The identification of predictive determinants of sensitivity may help to identify ependymoma subsets that could most likely benefit from BET inhibitor therapies.

Two reviews in the Special Issue are addressed to the analysis of current molecular targeting strategies in gliomas. In detail, Genoud and Migliorini focused their attention on immunotherapeutic approaches in glioblastoma [6]. Glioblastomas, the high-grade gliomas, are the most frequent and aggressive primary tumors of the central nervous system in adults, and no clear improvements over current the standard of care have been made in the last decade. Differently from other neoplasms and brain metastasis, immunotherapy has still not proven its efficacy in gliomas. Genaud and Migliorini reviewed the key glioblastoma immune-related features and the current immunotherapeutic strategies explored with their caveats [6]. Typical hallmarks of gliomas are molecular alterations of the RTK/PI3K/mTOR axis, and its targeting still represents a strong rationale for developing therapeutic strategies against gliomas. In their review article, Colardo et al. described the involvement of RTK expression and signaling in glioma models. RTK/PI3K/mTOR axis dysregulation has also been correlated to Temozolomide resistance, and preclinical and clinical studies targeting single or different components of the pathway are discussed [7].

Lastly, an interesting and original article was aimed at characterizing the exosomal DNA isolated from plasma of neuroblastoma (NB) patients and its potential use for detection of the mutational status of parental tumor cells [8]. Exosomes are small extracellular vesicles secreted by most cells, including tumor cells, playing a crucial role in cell–cell communication. Using an enzymatic method, Degli Esposti et al. provided evidence for the presence of double-stranded DNA in plasma-derived NB exosomes. They also showed, by whole-exome sequencing, that NB exo-DNA carries the tumor-specific genetic mutations of parental tumor cells, thus representing a powerful tool in the clinic; for these reasons, exosome DNA may represent an attractive non-invasive biomarker for NB molecular diagnostics [8].

Despite their diversity, nervous system tumors share the tendency of being refractory to radical surgical resection, and radio-chemotherapy is often ineffective due to resistance mechanisms. For these reasons, clinical management of these kinds of tumors is frequently challenging and the prognosis is still poor. In this scenario, precision medicine approaches, based on the continuous technological advances, are emerging as promising therapeutic avenues against nervous system tumors and highlight the need to acquire in-depth knowledge about the genetic mutations, signaling pathways, metabolic alterations, and environmental effects involved in tumor biology and resistance to therapies.
